# Supplementary material for: A Genetically Engineered Bivalent Vaccine Coexpressing a Molecular Adjuvant against Classical Swine Fever and Porcine Epidemic Diarrhea
Source: Int J Mol Sci. 2023 Jul 26;24(15):11954. doi: 10.3390/ijms241511954 (PMC10419043; doi:10.3390/ijms241511954)
Supplement: Supplementary file 1 [file ijms-24-11954-s001.zip › ijms-2490273-supplementary.pdf]

Supplementary materials

1. *Construction of eukaryotic expression plasmids for self-cleavage analysis of C-strain N^{pro} and its mutant N^{pro}_{D136N}*

To analyze the effect of the residue 136th mutation (D136N) located in N^{pro} on autoprotease activity, we constructed pN^{pro}-CoreEGFP, pN^{pro}_{D136N}-CoreEGFP and pN^{pro}_{D136N}CARD-CoreEGFP, respectively. Briefly, the fragment consisted of N^{pro} gene of the C-strain ~ 1-243 nt sequence encoding partial Core ~ sequence encoding linker (GGGGSGGGGS) ~ EGFP gene was synthesized by Sangon Biotech (Shanghai, China). The synthetic fragment was digested with *Afl* II and *Not* I. After purification of digested product, the purified fragment was linked into *Afl* II/*Not* I-digested pVAX1 to generate pN^{pro}-CoreEGFP. Similarly, the fragment consisted of N^{pro}_{D136N} gene (or sequence encoding N^{pro}_{D136N}CARD) ~ 1-243 nt sequence encoding partial Core ~ encoding linker (GGGGSGGGGS) ~ EGFP gene was synthesized, respectively. After digestion and purification, the purified fragment was linked into *Afl* II/*Not* I-digested pVAX1 to generate pN^{pro}_{D136N}-CoreEGFP or pN^{pro}_{D136N}CARD-CoreEGFP (Figure S1). All constructs were sequenced to confirm the identities.

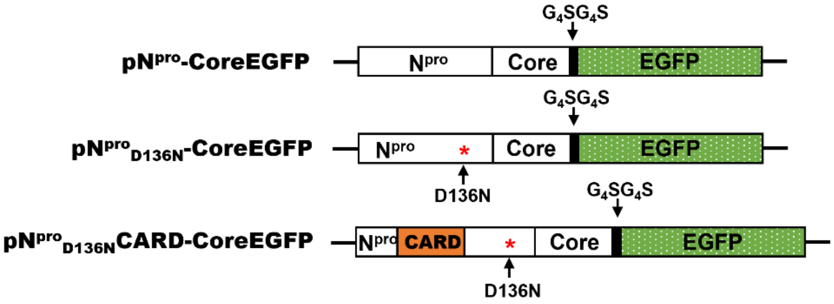

Figure S1. Schematics of the eukaryotic expression plasmids pN^{pro}-CoreEGFP, pN^{pro}_{D136N}-CoreEGFP and pN^{pro}_{D136N}CARD-CoreEGFP.

2. *Construction of the infectious cDNA clone of CSFV vaccine C-strain harboring the residue 136th mutation ($D_{136}N$)*

To construct the infectious cDNA clone of C-strain harboring D136N mutation, the N^{pro}_{D136N} fragment (**F_A**) was amplified by PCR using the plasmid pVAX1-N^{pro}_{D136N}-Flag as template with specific primers C292-F/C901-R, and the fragment covering core to E2 region (nucleotides 878 to 3243) (**F_B**) was amplified by PCR using pSPTI/C cDNA as template with primers C878-F/C3243-R (Table S1), respectively. After purification of the PCR products, a mixture of two fragments (**F_A** and **F_B**) were used as templates for overlap extension PCR with specific primers C292-F/C3243-R to generate the fragment **F_C**. After digestion with *Pml* I and *Nsi* I, the digested-fragment **F_C** was cloned into *Pml* I/*Nsi* I-digested pSPTI/C/SM3'UTR cDNA to generate the cDNA clone pC/SM3'UTR$_N$ (Figure S2). The construct was sequenced to confirm its identity.

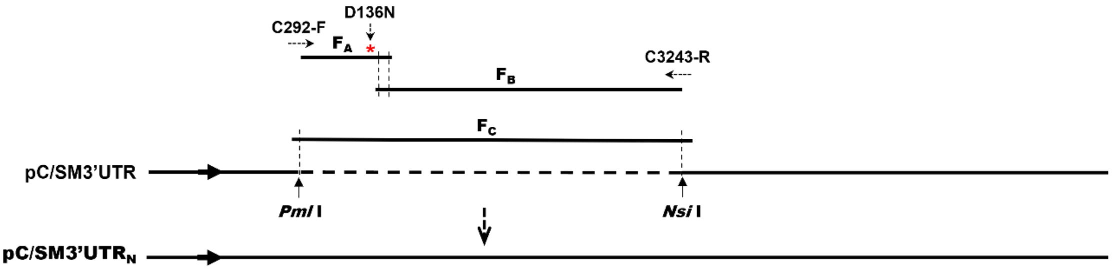

Figure S2. Schematics of the construction of the infectious cDNA clone of vaccine C-strain harboring the residue 136th ($D_{136}N$) (Red asterisk) using restriction enzyme-digested amplified fragments.

3. *Construction of the infectious chimeric cDNA clone of CSFV vaccine C-strain expressing foreign protein domains*

The chimeric cDNA clones of vaccine C-strain expressing foreign protein domains were constructed by following steps, respectively. Briefly, the N^{pro}_{D136N} fragment (**F1**) was amplified by PCR using the plasmid pVAX1-N^{pro}_{D136N}-Flag as template with specific primers C292-F/C901-R. A mixture of the purified **F1** and the synthetized S1NCOE/2A fragment (**F2**) was used as template for construction of the fragment **F12**. The core to E2 region (**F3**) were amplified by PCR using pC/SM3'UTR as template with primers 2Acore-F/C3243-R (**Table S1**). After purification of the PCR products, the fragment **F123** was amplified by an overlap extension PCR using a mixture of purified **F12** and **F3** as templates with primers C292-F and C3243-R. After digestion with restrict enzymes *Pml* I and *Nsi* I, the digested-F123 was cloned into *Pml* I/*Nsi* I-digested pC/SM3'UTR to generate the cDNA clone pC/SM3'UTRN-S1NCOE. To obtain the fragment tPAs/S1NCOE/2A (**F2′**) was amplified using the S1NCOE/2A as template by an overlapping PCR with primers tPAs-F/tPAs-R and tPAsS1N-F/2A-R (**Table S1**). Then, the fragment **F12′** was amplified using a mixture of the purified **F1** and **F2′** as template with primers C292-F and 2A-R. The fragment **F12′3** was amplified by PCR using a mixture of the **F12′** and **F3** as template with with primers C292-F and C3243-R. After digestion with

restrict enzymes *Pml* I and *Nsi* I, the digested-**F12′3** was cloned into *Pml* I/*Nsi* I-digested pC/SM3′UTR to generate the pC/SM3′UTR$_{N}$-tPAsS1NCOE. Similarly, the $N^{pro}{}_{D136N}$CARD fragment (**F1′**) was amplified by PCR using the plasmid pVAX1-$N^{pro}{}_{D136N}$CARD-Flag as template with specific primers C292-F/C901-R, then the fragment **F1′2′** was amplified using a mixture of the purified **F1′** and **F2′** as template with primers C292-F and 2A-R. The fragment **F1′2′3** was amplified by PCR using a mixture of the **F1′2′** and **F3** as template with primers C292-F and C3243-R. The digested-F1′2′3 was cloned into *Pml* I/*Nsi* I-digested pC/SM3′UTR to obtain pC/SM3′UTR$_{N}$-CARD/tPAsS1NCOE. All constructs were sequenced to confirm the identities.

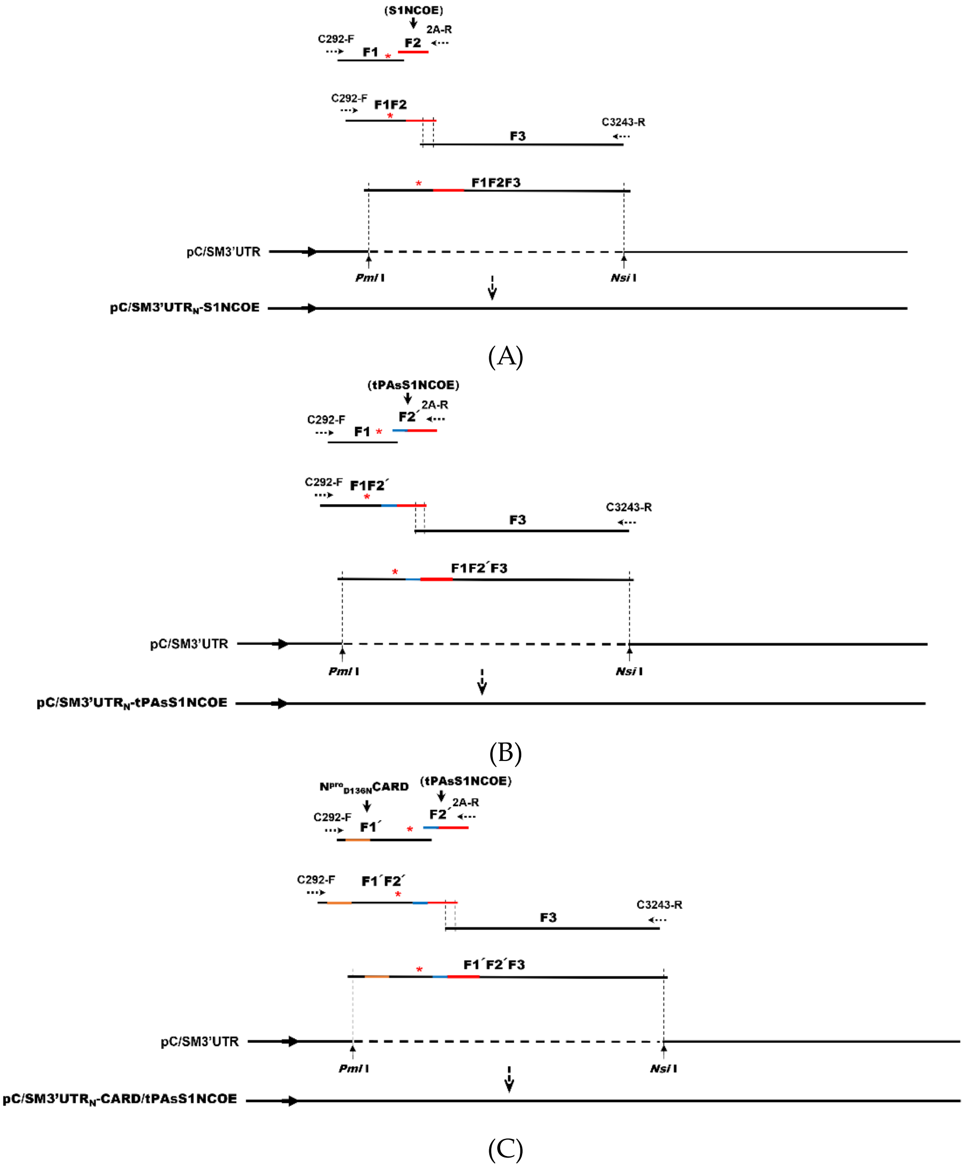

Figure S3. Schematics of the construction of the infectious cDNA clone chimeric C-strain using restriction enzyme-digested amplified fragments. **(A)** pC/SM3′UTR$_{N}$-S1NCOE; **(B)** pC/SM3′UTR$_{N}$-tPAsS1NCOE; **(C)** pC/SM3′UTR$_{N}$-CARD/tPAsS1NCOE.

Table S1. Primers for construction of recombinant plasmids and infectious cDNA clones

Primer	Sequence (5'-3')	Description
N^{pro}(SM)-F	CCGCTTAAG[a]ATGGAGTTGAATCATTTTGAACTTTTATAC (*Afl* II)	pVAX1-N^{pro}-C
N^{pro}(C)-F	CCGCTTAAGATGGAGTTGAATCACTTTGAACTTTTATAC (*Afl* II)	
N^{pro}-Flag-R	ATAAGAATGCGGCCGCCTTGTCATCGTCGTCCTTGTAATCGCA ACTGGTAACCCACAATGGACAG (*Not* I)	pVAX1-N^{pro}-SM
N^{pro}CARD-F	CCGCTTAAGATGGAGTTGAATCACTTTGAACTTTTATACAAAA CAAACATGACCTTCGCCGAA (*Afl* II)	pVAX1-N^{pro}CARD-Flag
CARD-R	CCCATTGGTTTTTGTTTGCTGTTGCGCAGCAG	
CARD-F	CTGCTGCGCAACAGCAAACAAAAACCAATGGG	
N^{pro}CARD-R	ATAAGAATGCGGCCGCCTTGTCATCGTCGTCCTTG (*Not* I)	
D136N-F	CATATGTGTGCATC*AAT*[b]GGCTGC	pVAX1-N^{pro}_{D136N}-Flag
D136N-R	GCAGCC*ATT*GATGCACACATATG	pVAX1-N^{pro}_{D136N}CARD-Flag
C292-F	CGCACGTGATGGGAGTACGACCTGATAGGGCGCTGCAGAGGC CCACTATTAGGCTAGTATAAAAATCTCTGCTGTACATGGCACA TGGAGTTGAATCACTT (*Pml* I)	pC/SM3′UTR_N
C901-R	CTCCCACTCGCGCCATCATCGGAGCAACTGGTAACCCACAAT GG	
C878-F	TCCGATGATGGCGCGAGTGGG	
C3243-R	CCAATGCATGCACCTTGACAGTCGTGTTACCGATCAAGCAC (*Nsi* I)	
tPAs-F	TCCGATGATGGCGCGAGTGGGATGGATGCAATGAAG	pC/SM3′UTR_N-S1NCOE
tPAs-R	GCACCTGGTGACATCTGAAGAGATTTCGC	pC/SM3′UTR_N-tPAsS1NCOE
tPAsS1N-F	GCGAAATCTCTTCAGATGTCACCAGGTGC	
2A-R	AGGCCCAGGGTTCTCCTCCACGTC	pC/SM3′UTR_N-CARD/tPAsS1NCOE
2Acore-F	GTGGAGGAGAACCCTGGGCCTTCCGATGATGGCGCGAGTG	

[a] Corresponding restriction enzyme sites are underlined;
[b] Mutation sites are indicated in italics.

Table S2. Specific primers for qRT-PCR

Primer	Sequence (5'-3')	Descrpition
NS5B-F	ATCTGCCTACAAGGAAGTCATCGG	CSFV
NS5B-R	CCAGTTGCCCTCTTTAACACCCATA	
N-F	CGCAAAGACTGAACCCACTAACCT	PEDV
N-R	TTGCCTCTGTTGTTACTTGGAGAT	
